# Hospitals and Insurance Systems

**Published:** 1910-10-01

**Authors:** H. P. Bosscha

**Affiliations:** Utrecht, Holland.


### HOSPITALS AND INSURANCE SYSTEMS.

By Dr. H. P. BOSSCHA, Utrecht, Holland.

Compulsory insurance against disease, accidents, and sickness generally, notwithstanding the fact that it is a comparatively modern innovation, dating back only a couple of decades, has exercised a vast influence for good upon the relief of sickness in the German Empire. To a very large extent, too, it has had an important influence upon the development of the hospital system in other countries. The actual insurance against disease, or against its results, as a system has contributed far less to the establishment of special institutions than other forms of insurance, but no other system of insurance, it may safely be premised, has had so important and far-reaching an influence upon the development of the general hospital system. Grotjahn, who has dealt with this subject in detail, goes so far as to postulate that this system of insurance initiated the movement which made it possible for the better class working-man to enter the hospitals. That may have been the case so far as Germany is concerned—a matter which I will not discuss—but it certainly is not the case with Holland or other countries where the hospital system is well organised. At the same time it is only fair to say that, at the time when under the influence of the social legislation in Germany important modifications of the hospital population were effected in that country, a somewhat similar though by no means so conspicuous nor yet so powerful elevation of the social condition of the hospital population took place in this country (Holland). In other words, the hospital has become more popular with the public, and this popularity is likely to be considerably increased in the event of the universal introduction of compulsory insurance against disease.

In order to grasp the significance of the subject and to judge of the importance of the influence exercised by insurance systems upon the development of hospitals generally it is necessary to pay some attention to the state of affairs in Germany, always bearing in mind that the conditions in other countries are in many respects entirely different from those obtaining there. A review of the German provisions leads one to conclude that the most important legislative enactments are as follows:—

Firstly, that the decision with regard to the admission into hospital is vested in the sick fund authorities, and not in the medical man himself. Fortunately, in Germany, in practice it is found that the actual decision usually rests with the physician, whatever the law may say.

Secondly, that while originally strict regulations governing the admission of patients into hospital have been made with a view to preventing imposition by malingerers and chronics, the actual working brought about a state of affairs wholly different from that contemplated by the legislature.

Thirdly, that the patient—that is, the sick person—has no right to claim hospital treatment. Grotjahn declares that this omission in the law is due to the fact that it was impossible at the time when it was enacted to acknowledge the right to hospital treatment, in view of the fact that the existing hospital accommodation was manifestly insufficient. It appears to me that it is extremely undesirable to copy the German example in this respect, even though hospital accommodation may as yet be admittedly inadequate.

Fourthly, that those policyholders who possess families to be supported out of their wages may, in addition to hospital treatment, receive sick money to the extent of half their ordinary wage, while policyholders who have no family to support may receive sick money calculated at a fourth of their ordinary rate of wages. It is chiefly this regulation that has made the hospitals so popular with the German working-man.

### Some Details of Finance.

To what extent the German sick funds avail themselves of these provisions, and, above all, of the hospitals, for the treatment of their sick members is easily seen from the statistical tabulations and from the statements of contributions to the various hospitals for sick fund purposes. These latter amounted for the whole German Empire to 10,425,000 marks in 1892, 11,569,000 in 1893, 11,868,800 in 1894, 12,604,100 in 1895, 13,575,000 in 1896, 14,804,000 in 1897, 15,852,300 in 1898, 17,883,800 in 1899, 19,607,800 in 1900, 20,641,200 in 1901, 21,343,000 in 1902, 23,658,800 in 1903, and 27,694,300 in 1904. The figures per member for sickness in general and for hospital treatment were as follows:—

| Year | For Sickness in General | For Hospital Treatment | Year | For Sickness in General | For Hospital Treatment |
|---|---|---|---|---|---|
| | Mark | Mark | | Mark | Mark |
| 1892 | 13.55 | 1.50 | 1899 | 15.87 | 1.95 |
| 1893 | 14.35 | 1.63 | 1900 | 16.58 | 2.06 |
| 1894 | 13.67 | 1.63 | 1901 | 16.94 | 2.14 |
| 1895 | 13.93 | 1.67 | 1902 | 17.02 | 2.16 |
| 1896 | 13.81 | 1.71 | 1903 | 17.69 | 2.31 |
| 1897 | 14.45 | 1.78 | 1904 | 19.97 | 2.59 |
| 1898 | 14.60 | 1.81 | | | |

A comparison between the sums expended on hospital treatment and other expenses sanctioned by the funds is interesting. From the table supplied it is shown that every 100 marks expended by the sick funds was apportioned as follows:—

| Year | Doctor | Chemist | Sick Money | Convalescent Fund | Midwifery | Death | Hospital Treatment |
|---|---|---|---|---|---|---|---|
| 1888 | 20.34 | 16.16 | 47.11 | | 1.32 | 4.27 | 10.80 |
| 1889 | 20.59 | 16.59 | 46.30 | | 1.29 | 4.07 | 11.16 |
| 1890 | 19.97 | 16.88 | 47.46 | | 1.21 | 3.90 | 10.58 |
| 1891 | 20.03 | 16.70 | 46.94 | | 1.32 | 3.71 | 11.30 |
| 1892 | 20.23 | 17.02 | 46.63 | | 1.29 | 3.77 | 11.06 |
| 1893 | 20.01 | 17.35 | 44.89 | 0.04 | 1.61 | 3.75 | 11.35 |
| 1894 | 22.30 | 17.50 | 42.78 | 0.07 | 1.78 | 3.65 | 11.92 |
| 1895 | 22.08 | 17.30 | 43.27 | 0.05 | 1.74 | 3.54 | 12.02 |
| 1896 | 22.61 | 17.23 | 42.35 | 0.07 | 1.84 | 3.53 | 12.37 |
| 1897 | 22.34 | 17.18 | 42.93 | 0.06 | 1.80 | 3.40 | 12.29 |
| 1898 | 22.73 | 17.19 | 42.47 | 0.07 | 1.83 | 3.33 | 12.38 |
| 1899 | 21.96 | 16.90 | 43.74 | 0.07 | 1.68 | 3.34 | 12.31 |
| 1900 | 21.75 | 16.47 | 44.31 | 0.07 | 1.62 | 3.36 | 12.42 |
| 1901 | 21.82 | 16.03 | 44.68 | 0.08 | 1.60 | 3.15 | 12.64 |
| 1902 | 22.35 | 15.84 | 44.33 | 0.08 | 1.62 | 3.05 | 12.72 |
| 1903 | 22.54 | 15.98 | 43.75 | 0.00 | 1.58 | 2.98 | 13.08 |
| 1904 | 22.40 | 15.02 | 44.77 | 0.07 | 2.00 | 2.79 | 12.95 |

The rate per head for hospital treatment rose from 1.63 mark in 1893 to 2.59 in 1904, an increase of 59 per cent.

### How the Funds Affect Hospitals.

The influence of the sick funds upon the hospitals has been unquestionable. While they opened the hospitals to the working-man, at the same time they favourably influenced the hospitals themselves by robbing the latter largely of their character as pauper asylums. At the same time they rendered these institutions more popular, and also robbed them of their reputation, undeserved but none the less prevalent, of being merely antechambers to death. It was increasingly recognised that the hospitals did good work. Gradually the old lugubrious popular idea of these institutions was dispelled, and it became known that they were excellent and deserving of all support.

Grotjahn thinks that this popularity of the hospitals is steadily increasing, and that in the future the working-man will devote more attention to these institutions and support them more actively than he does at present. He points out that in the table just given the *pro capita* rate of expenses for physician and chemist is far too high in comparison with that for hospital treatment, and deems it in the interest of the sick funds that this comparison should be so in the future that the main expenses will be devoted to hospital treatment *plus* sick fund support for the policy-holder's family. In connection with this it is advisable that the sick funds should be given a voice in the management of the affairs of the hospitals which they support in order to encourage mutual co-operation and friendliness.

### Separatist Tendencies.

It is in discussing this question of allowing the funds a say in the management of the affairs of the institutions that we have to consider a question which is of vital importance not only in Germany, but much more so, in fact, in those countries where compulsory insurance is not yet in being. As soon as the expenses of hospital treatment have risen above a certain figure, the large sick funds begin to moot the idea of a separate hospital of their own. Such a special hospital, they argue, will give them a free hand; they will possess full control over it, and consequently full control over the large sums which their treasuries have to expend under the heading of hospital treatment.

In Germany this tendency has so far remained an aspiration, and no actual steps have been taken to realise the wishes of the sick funds to possess their own and independent hospitals, where their members could be treated. This result is largely due to the fluctuation in the number of members of the various funds which has made it difficult for any one of them to undertake the heavy financial responsibility of founding and endowing a separate hospital, but also to a certain extent to the difference between the actual cost of hospital treatment and the sum allowed by the funds for such treatment, which in nearly all German hospitals is still too large to encourage separatist aspirations. This can be seen from the latest figures, as given in the *Taschenbuch für Krankenanstalts Betrieb*:—

| | Daily Cost per Patient. | Payment per Patient (ordinary). | Payment per Patient (Sick Fund rate). |
|---|---|---|---|
| | Marks. | Marks. | Marks. |
| Altona Städt. Krankh. ... | 4.30 ... | 2.59 ... | 2.50 |
| Augsburg „ „ ... | 2.54 ... | 2.36 ... | 2.50 |
| Barmen „ „ ... | 3.18 ... | 2.28 ... | 2.0 |
| Berlin Friedrichsh. ... | 3.67 ... | 1.53 ... | 2.50 |
| „ Moabit ... ... | 3.49 ... | 1.57 ... | 2.50 |
| „ Urban ... ... | 3.52 ... | 1.82 ... | 2.50 |
| „ K. U. K. Friedrich. Kinderkrankenhaus | 4.75 ... | 0.63 ... | 2.50 |
| Braunschweig Herzl. Kh.... | 3.49 ... | 2.08 ... | 2.20 |
| Charlottenburg Städt. Krh. | 4.50 ... | 2.56 ... | 2.50 |
| Dresden. Friedrichst. ... | 3.75 ... | 2.22 ... | 2.50 |
| „ Johannst. ... | 3.53 ... | 2.43 ... | 2.50 |
| Frankfurt a/M. Städt. Krh. | 4.45 ... | 2.24 ... | 1.75 |
| Hannover „ „ | 4.33 ... | 2.85 ... | 2.50 |
| Leipzig „ „ | 3.94 ... | 2.01 ... | 1.75 |
| München r. I. „ „ | 3.85 ... | 3.10 ... | 2.50 |
| „ l. I. „ „ | 3.93 ... | 2.90 ... | 2.50 |
| Nürnberg „ „ | 4.23 ... | 3.02 ... | 3.0 |
| Posen „ „ | 3.42 ... | 1.74 ... | 2.0 |
| Stettin „ „ | 4.32 ... | 1.90 ... | 2.0 |

With the exception of Augsburg, it will be seen that the actual cost of hospital treatment is in excess of the allowance made by the sick funds for such treatment in all these hospitals. That this tendency has been checked before the separatist movement has actually materialised, is exceedingly fortunate. Specialisation, so far as hospitals are concerned, is only justifiable on strictly medical or scientific grounds, and every policy which favours the establishment of special institutions for other objects or on other grounds is likely to damage both the hospitals themselves and the interests of the patients. It is to be hoped, therefore, that no definite demand will be made that sick funds should contribute sums proportionate to the actual cost per bed, since such attempts must inevitably encourage the desire to establish a separate and independent institution. Compulsory insurance can become the goose that lays the golden eggs for our hospitals, and it is sincerely to be hoped that no premature and foolish attempt will be made to kill the bird.

Turning to the somewhat analogous case of insurance against accident, it is to be noted that in Germany the sick funds, or rather accident funds, have already materialised this separatist aspiration and established independent casualty departments. The existence of such institutions is scarcely to be defended, and it is much rather to be wished that the funds should devote their energies to providing those who are incapacitated by accident with opportunities for performing productive labour.

With regard to insurance against invalidity, it is curious to note that this form of insurance has had a marked influence upon the development of hospitals in Germany, and that, moreover, although there is no mention whatever of hospital treatment in the first law of 1889. Yet soon after the passage of this law a patient was sent to the tuberculosis sanatorium at Gorbersdorf in order to obviate in this manner an early invalidity, an example which was rapidly followed until 1899, when the law was altered so as to pronounce the insurance companies capable of utilising institutional treatment to prevent invalidity. How far the funds have availed themselves of this clause is readily seen from the following figures:—

| Year | Patients | Expenses | Percentages Contribution |
|---|---|---|---|
| 1898 | 13,758 | 2,769,430 | 2.1 |
| 1899 | 20,039 | 4,056,975 | 2.8 |
| 1900 | 27,427 | 6,210,720 | 4.0 |
| 1901 | 32,710 | 7,912,219 | 4.9 |
| 1902 | 35,949 | 9,056,240 | 5.4 |
| 1903 | 43,593 | 11,501,205 | 6.5 |
| 1904 | 49,421 | 12,735,081 | 6.6 |
| 1905 | 56,620 | 14,448,005 | 7.1 |

A large percentage of these patients—54 per cent.—were sufferers from phthisis, and the following additional figures are, therefore, of some interest:—

| | Expenses | Cost per Head of Cases Successfully Treated | Cost per Head of Unsuccessful Cases |
|---|---|---|---|
| Phthisis patients | 9,679,535 | 394 | 225 |
| Other patients | 4,483,192 | 209 | 163 |

The funds expended for these objects have naturally benefited the sanatoria. It is, of course, impossible to say at this stage in how far the establishment of prophylactic institutional treatment has justified itself.

Taking, then, a broad survey of the insurance system, so far as its working in Germany is concerned, we are able to draw the following conclusions: (1) The social insurance laws have had a large influence upon the development of the hospitals in Germany and have been mainly responsible for popularising these institutions. (2) In particular these laws and the system of insurance generally have been responsible for improving the condition of the hospitals and for robbing them of their character of pauper asylums. (3) The system of insurance against accidents has had an influence upon the development of the casualty and surgical departments of the general hospitals, and has largely contributed to the modern surgical tendency which strives to obtain not merely anatomical, but perfect functional cures in cases of accidents. (4) The system of insurance against invalidity has greatly influenced prophylactic institutional treatment, especially in cases of pulmonary disease. (5) It is desirable to adopt measures to counteract the tendency on the part of insurance societies to establish their own hospitals, since the specialisation of hospitals ought to be regulated by the nature of the diseases to be treated, and not by the policy or wishes of the supporters of such institutions.

